# Industry Upgrading: Recommendations of New Products Based on World Trade Network

**DOI:** 10.3390/e21010039

**Published:** 2019-01-09

**Authors:** Wen-Yao Zhang, Bo-Lun Chen, Yi-Xiu Kong, Gui-Yuan Shi, Yi-Cheng Zhang

**Affiliations:** 1 Faculty of Computer and Software Engineering, Huaiyin Institute of Technology, Huaian 23303, China; 2 Department of Physics, University of Fribourg, 1700 Fribourg, Switzerland

**Keywords:** economic complexity, proximity, industry upgrading

## Abstract

GDP is a classic indicator of the extent of national economic development. Research based on the World Trade Network has found that a country’s GDP depends largely on the products it exports. In order to increase the competitiveness of a country and further increase its GDP, a crucial issue is finding the right direction to upgrade the industry so that the country can enhance its competitiveness. The proximity indicator measures the similarity between products and can be used to predict the probability that a country will develop a new industry. On the other hand, the Fitness–Complexity algorithm can help to find the important products and developing countries. In this paper, we find that the maximum of the proximity between a certain product and a country’s existing products is highly correlated with the probability that the country exports this new product in the next year. In addition, we find that the more products that are related to a certain product, the higher probability of the emergence of the new product. Finally, we combine the proximity indicator and the Fitness–Complexity algorithm and then attempt to provide a recommendation list of new products that can help developing countries to upgrade their industry. A few examples are given in the end.

## 1. Introduction

Understanding the economic situation of a country or region is crucial to the formulation of both macroeconomic policies and microscopic investment decisions. GDP, a classic monetary indicator, has been a good measure of the state of the economy. However, the accurate GDP depends on a thorough investigation across many different sectors of economy and thus lacks predictive power. Therefore, a new metric to predict the future GDP of the economy and its growth has practical significance.

As both the international trade and the trade volume continue to increase, the impact of the international trade sector on national economy rises over the past few decades. The World Trade Network has attracted the attention of researchers in many fields and has become an important research field in studying the economic development of countries. By studying the structure and dynamics of trade networks, physicists have made it possible to explain the state of development and potential of the country’s economy from the complex interactions among nations [1]. Hausmann and Hidalgo et al. [2,3] proposed the Economic Complexity Index (ECI) to measure diversification of a country and the ubiquity of a product. The facts show that ECI has a strong correlation with the growth of national GDP. Similarly, Tacchella et al. [4,5,6] proposed a new fitness–complexity algorithm that defines the country’s fitness and product’s complexity—by finding the fixed points of a set of nonlinear iterative equations. Pugliese et al. [7] and Wu et al. [8] further studied the convergence and stability of the algorithm. Cristelli et al. [9] draw the countries’ fitness and GDP per capita onto a fitness–income plane: the countries with high fitness which export multiple complex products have strong competitiveness, while, for the countries with low fitness, the industry structure is relatively simple, and GDP tends to be greatly fluctuating over time. In addition, they also identify a ‘laminar region’ where the countries inside have high fitness but low GDP per capita. The results show that these countries tend to have a constant fast growth of GDP per capita in the next few years. On the other hand, the countries with low fitness encounter a chaotic situation that the growth rate of economy is very unstable and unpredictable. The fitness of countries revealed by the algorithm demonstrates its ability to predict long-term GDP growth, and scientists apply it to make economic forecasts in the country [10] and the region [11].

In addition to predicting the country’s GDP growth, a more meaningful research topic for the underdeveloped countries is how to improve national competitiveness by making suitable macroscopic industrial upgrading plans in order to escape the poverty trap. In the year of 2007, Hidalgo et al. [12] proposed a new metric called “proximity” to measure the spatial distance between products in the product space constructed by the World Trade Network. Consequently, the emergence of new products can be approximated as the result of the spreading process of existing products in the product space, which suggests that new products that have closer distance (higher similarity) with existing products are easier to be developed in the future. Recently, Alshamsi et al. [13] discovered that the probability that a country will export a product more than 25% of the world average per capita is positively correlated with the fraction of related products already exported by that country. Vidmer et al. [14] used several recommendation system methods to study the emergence of new products in the world trade network, and found that the recommended products will have higher probability to appear in the following years. All the results indicated that there are certain tendencies for the emergence of new industries, and some specific products are more likely to appear in the basket of the country’s new exports.

In this paper, we combine the proximity indicator and the fitness–complexity algorithm to study what industries can possibly improve a country’s fitness. First, we use the “proximity” metric to find the products that a country is capable of developing. Furthermore, we define a list of “core products” which not only have high export volumes, but also are complex products that have a relatively high complexity. These “core products” are regarded as the target products to enhance the country’s fitness. Finally, we attempt to recommend the relevant products for the developing countries who have a higher probability to produce according to the proximity of the product space.

## 2. Results

### 2.1. Prediction Ability of Proximity

We start with calculating the proximity between products and obtain the proximity matrix ϕ, an element $\phi_{p_{i},p_{j}}$ represents the similarity between product $p_{i}$ and product $p_{j}$ (details can be found in Materials and Methods). We further define a matrix $\phi^{max}$, an element $\phi_{c,p}^{max}$ that represents the maximal proximity between *p* and all the products that country *c* already exports (if country *c* already exports *p*, we set $\phi_{c,p}^{max}$ to be a number larger than 1 for convenience of computation):

$$\phi_{c,p}^{max}=max_{i}\left(M_{c,p_{i}}\phi_{p_{i},p_{j}}\right).$$

Here, *M* is the binary country–product matrix, an element $M_{c,p_{i}}$ is 1 if the country *c* export product $p_{i}$ and 0 otherwise (see Materials and Methods for details).

**Figure 1:**
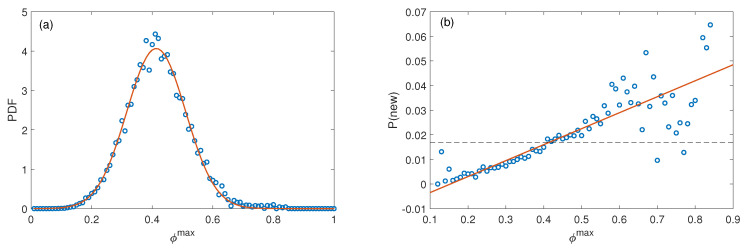
(**a**) distribution of $\phi_{max}$ of products that a country has not yet developed; (**b**) probability that a country develops a new product in the next year versus $\phi_{max}$ of the new product. The dashed line represents the average probability to develop a new product. The large fluctuations at both ends are due to the relative small number of the denominator $N_{i}$.

We use this method to analyze the World Trade Network data from 2001 to 2014 (see Materials and Methods for details). If a country exports a product that has never been exported by the country, we consider this product as a new product that the country develops during the year. We firstly study whether the probability of a new product’s occurrence is related to the $\phi^{max}$ of this product, which means whether the maximal similarity of products is related to the probability of the new product’s emergence. To verify, we divide the $\phi^{max}$ into 100 groups from [0, 0.01] to (0.99, 1] (note that the products the country already exports will not be included since we set it to be larger than 1), and count the number of products in each group. The *i*th group consists of $N_{i}$ products. The distribution of $\phi^{max}$ follows a normal distribution, as shown in Figure 1a.

Similarly, we count the number of products that appear in the next year for each group *i*, denoted by $n_{i}$. We finally obtain the empirical probability that a product *p* with $\phi_{c,p}^{max}$ that to be developed by country *c* in the next year, $P{\left(new\right)}_{i}=n_{i}/N_{i}$. We can observe a significant positive correlation between P(new) and $\phi^{max}$, as shown in Figure 1b, which suggests that, if a product is very similar (high proximity) to a product a country already exports, the country will have a relatively high probability to develop this new product.

**Figure 2:**
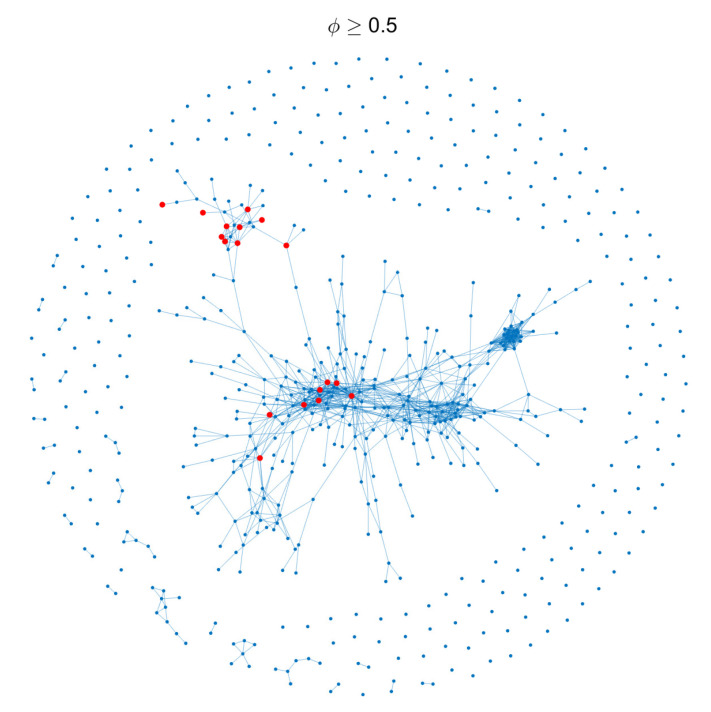
The product space for threshold ϕ≥0.5; each edge links two products that have a proximity larger than 0.5. The red nodes represent the core products which we define later.

In addition, from a conventional perspective, the more relevant industries a country has, the more probable for the country to develop the new product. As one can imagine, if a country has many products that are quite similar to an unprecedented product, the country must be more developed in the relevant industries. We will then quantitatively analyze this correlation. We firstly plot the product space network, in which each edge between two products represents the two products have a proximity larger than ϕ=0.5, as shown in Figure 2.

**Figure 3:**
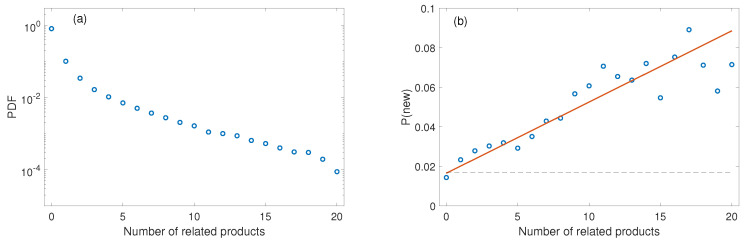
(**a**) distribution of number of related existing products for a new product; (**b**) probability that a country develops a new product in the next year versus number of related existing products. The dashed line represents the average probability to develop a new product. The straight line indicates the linear fit.

We then count the number of existing products that is related to the unprecedented products of a country. The distribution can be found in Figure 3a. The relation of the probability that a country develops a new product in the next year and number of related existing products is shown in Figure 3b. The significant positive correlation between the probability and the number of related existing products suggests that the more correlated products (which has a high proximity with the target product) a country exports, the larger probability the target product can be developed.

### 2.2. Recommending New Products to Countries

Through the above analysis, we can conclude that some new industries are more probable to be developed by a country than other industries. The probability is related to both the maximal proximity between the country and the product, and the number of related industries that the country already has. Among all the possible industries, which are the most important ones? Next, we will combine the fitness–complexity algorithm to find a set of products that have both high trading volume and high complexity. A large trading volume is crucial to improving the income of a country, and the complexity of a product will enhance the fitness of a country in the international competition. We define these products as “core products” and use them as the target of recommendation for the developing countries.

We firstly give the list of core products. Here, we simply choose the products who rank in the top 100 in the Complexity measure (see Appendix A) and the top 100 in the gross export volume, at the same time having neighbors in the product space that enable us to find the possible related existing products.

We obtain a final list consists of 18 products, including Glycosides and Vaccines, Motor Vehicles Piston Engines, Piston Engine Parts, Machinery for Specialized Industries, Miscellaneous Heating and Cooling Equipment, Pulley System Parts, Miscellaneous Office Equipment, CPUs, Computer Peripherals, Computer Parts and Accessories, Color TVs, Telecom Parts and Accessories, Printed Circuits, Miscellaneous Electronic Circuit Parts, Automotive Electrical Equipment, Vehicles Parts and Accessories, Lighting Fixtures, Optical Lenses. Their neighbors in the product space can be found in Appendix A.

**Figure 4:**
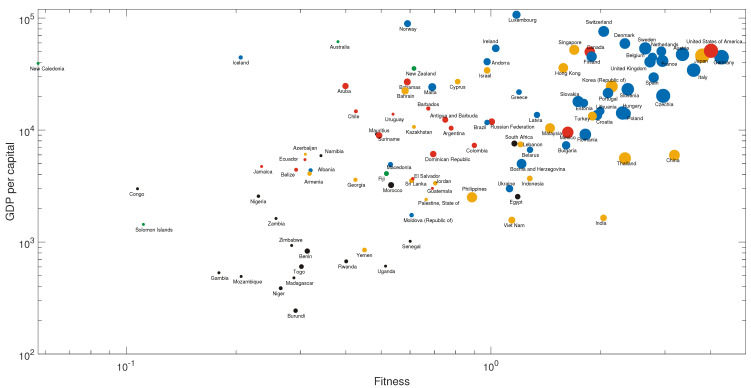
The overview of fitness–income plane of countries (regions). The size of dots represents the sum of the number of related existing products for all core products. The color of dots: blue, yellow, black, green, and red, represent European, Asian, African, Oceanian and American countries (regions), respectively.

We then sum up the number of related existing products for all core products as a measure of the potential capability of a country to develop the core products, as shown in Figure 4. We choose Burundi as an example of low-income countries for later recommendation in Table 1.

**Figure 5:**
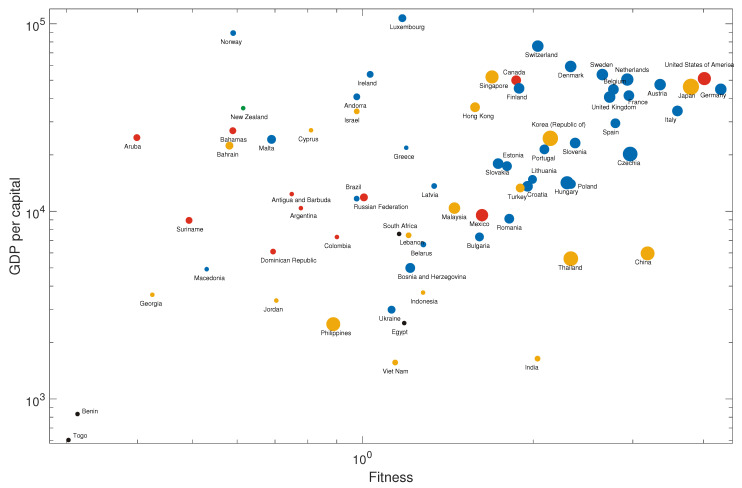
The overview of fitness–income plane of countries (regions). The size of dots represents the number of core products to which the country (region) has at least three related products. The color of dots: blue, yellow, black, green, and red, represent European, Asian, African, Oceanian and American countries (regions), respectively.

From the idea that the products that have more links to a country will be easier for that country to develop, we count the number of core products that have at least three links to a country, as shown in Figure 5.

Togo and the Philippines are chosen as exemplary countries for recommendation because Togo lies in the left bottom corner of the panel, which means that it has both low income and low fitness, and the Philippines has relatively more related core products. Thus, we think this country has a high potential to develop new industries. The recommendations to the three countries—Burundi, Togo, and the Philippines—are given in Table 1.

## 3. Discussion

In this paper, we combine the fitness–complexity algorithm and the proximity indicator to recommend industry upgrading path to developing countries. Firstly, we find the maximal proximity of a certain product and the existing products of a country have a strong positive linear correlation with the probability that the country produces the new product in the future. Furthermore, we find that the probability that a country develops a new product also increases with the number of existing industries of a country that are related to the industry. Based on the two discoveries above, we conclude that the proximity can be used to find the easily accessible industries for a country to develop. Combined with the Complexity metric, we define a set of target products, which we call the “core products”, that can improve both the fitness and income of the country. Using the above method, we recommend the industry upgrading road maps for three countries to develop these core products as examples. In this paper, we only consider the recommendation of the directly related products. The recommendation on indirectly related products that need multi-step development is still open for future research.

## 4. Materials and Methods

### 4.1. Data Description

**Figure 6:**
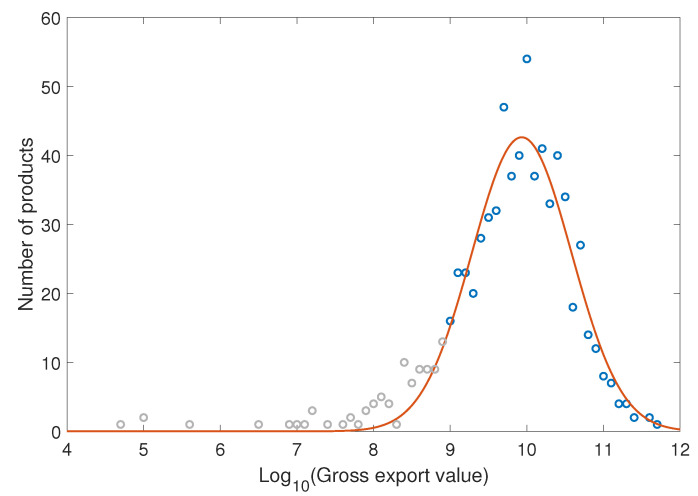
The distribution of export volume. Note that it fits well with log-normal distribution.

The dataset we use for our analysis are the World Trade Network dataset provided by United Nations COMTRADE [15] and the GDP stats from National Accounts Main Aggregates Database [16]. In the World Trade Network dataset, the products whose classification code ended with letters A and X or a 0 are those who are not in the official classification, we dump those data in our study. In addition, we only used the information of countries that exist in the two datasets simultaneously. Furthermore, the products with trading volume fewer than 1 billion US dollars are excluded. In Figure 6, we show the distribution of export volume of the products. The products in gray area are excluded in our research. After the data cleaning, the dataset contains the trading information of 130 countries and 619 products from 2001 to 2014.

### 4.2. Revealed Comparative Advantage

To determine the entries of trading network, the Revealed Comparative Advantage (RCA) [17] is used to calculate whether one country has a link with a product. The RCA is defined as:

(1) $$RCA_{i,\alpha }=\frac{e_{i,\alpha }}{\sum_{\beta }e_{i,\beta }}/\frac{\sum_{j}e_{j,\alpha }}{\sum_{j,\beta }e_{j,\beta }},$$

where $e_{i,\alpha }$ is the volume of products α exported by country *i* in thousands of US dollars. RCA describes the relative importance of a country’s specific exporting product, compared to the product exports by all other countries. We use a bipartite network representation with two different types of nodes: one for the country and one for the product. All country–product pairs with a higher than RCA threshold—set to 1—are therefore connected by a link between the corresponding nodes in the bipartite network, denoted by $M_{c,p}$.

To avoid the large fluctuations of complexity and proximity in different years here, we bundle the data of year 2001–2014 together, and the RCA Matrix used for calculation of complexity and proximity is given below:

$$\left[\begin{array}{c} RCA_{2001}\\ RCA_{2002}\\ ...\\ RCA_{2014} \end{array}\right].$$

### 4.3. Proximity

One important metric in this paper is the proximity proposed by Hidalgo [12]. The main idea of proximity comes from that in the evolution process of industry upgrading. Fewer attempts exist when the two industries are widely separated. Proximity is to assess the distances between different products in the “product space”. In Ref. [12], the proximity $\phi_{i,j}$ of products $p_{i}$ and $p_{j}$ is given by:

(2) $$\phi_{i,j}=min\left\{P\left(M_{c,p_{i}}|M_{c,p_{j}}\right),P\left(M_{c,p_{j}}|M_{c,p_{i}}\right)\right\},$$

where $P(M_{c,p_{i}}|M_{c,p_{j}})$ is the probability that a country exports $p_{i}$ given that the country exports $p_{j}$.

### 4.4. Fitness–Complexity Algorithm

Economic complexity is an indicator that assigns scores to individual countries and products. Instead of describing the relationship between products, in Ref. [4], the country-product network uses a set of self-consistent equations to study the country’s fitness and product complexity. The fitness of a country indicates its ability to manufacture complex products relative to other countries, and the complexity of the product indicates the amount of technology required to produce it. Country fitness and product complexity are defined as

(3) $$\begin{array}{cccc} F_{c}^{\left(0\right)} & =1\;\forall c, & Q_{p}^{\left(0\right)} & =1\;\forall p,\\ {\tilde{F}}_{c}^{\left(n\right)} & =\sum_{p}M_{cp}Q_{p}^{\left(n-1\right)},\; & {\tilde{Q}}_{p}^{\left(n\right)} & =\frac{1}{\sum_{c}M_{cp}\frac{1}{F_{c}^{\left(n-1\right)}}},\\ F_{c}^{\left(n\right)} & =\frac{{\tilde{F}}_{c}^{\left(n\right)}}{{\langle {\tilde{F}}_{c}^{\left(n\right)}\rangle }_{c}}, & Q_{p}^{\left(n\right)} & =\frac{{\tilde{Q}}_{p}^{\left(n\right)}}{{\langle {\tilde{Q}}_{p}^{\left(n\right)}\rangle }_{p}}, \end{array}$$

where *n* is the current iteration, $F_{c}^{\left(n\right)}$ is the fitness of country *c* at iteration step *n* and $Q_{p}^{\left(n\right)}$ is the complexity of product *p* at step *n*. Fitness and complexity are initialized as $F_{c}^{\left(0\right)}=Q_{p}^{\left(0\right)}=1$ and normalized after each iteration so that their sum is *N* and *M*, respectively. $M_{c,p}$ is the matrix of RCA we use. 

## Figures and Tables

**Table 1:** Three exemplary cases of recommendation to countries. The left side shows the related existing industries, and the right side shows the corresponding recommended core industries.

Existing Industries	Predicted Industries
**Recommendation list for Burundi:**	
Parts of Metalworking Machine Tools	Pulley System Parts
Video and Sound Recorders	Miscellaneous Office Equipment
Video and Sound Recorders	Computer Peripherals
Parts of Metalworking Machine Tools	Automotive Electrical Equipment
Parts of Metalworking Machine Tools	Vehicles Parts and Accessories
**Recommendation list for Togo:**	
Miscellaneous Engines	Miscellaneous Heating and Cooling Equipment
Lifting and Loading Machinery	
Miscellaneous Engines	Pulley System Parts
Seamless Iron Tubes	Vehicles Parts and Accessories
Miscellaneous Engines	
Lifting and Loading Machinery	
**Recommendation list for Philippines:**	
Printing Machine Parts	Glycosides and Vaccines
Miscellaneous Centrifuge and Filtering Machinery	Motor Vehicles Piston Engines
Non-Electrical Counting Devices	
Control Instruments of Gas or Liquid	
Miscellaneous Centrifuge and Filtering Machinery	Piston Engine Parts
Control Instruments of Gas or Liquid	
Miscellaneous Metalworking Machinery	Machinery for Specialized Industries
Miscellaneous Engines	Pulley System Parts
Parts of Metalworking Machine Tools	
Miscellaneous Metalworking Machinery	
Miscellaneous Heating and Cooling Equipment	
Miscellaneous Centrifuge and Filtering Machinery	
Roller Bearings	
Computer Peripherals	CPUs
Vehicles Stereos	Color TVs
Computer Peripherals	Telecom Parts and Accessories
Vehicles Stereos	
Miscellaneous Power Machinery	
Printed Circuits	
Miscellaneous Electrical Machinery	
Computer Peripherals	Miscellaneous Electronic Circuit Parts
Computer Parts and Accessories	
Printed Circuits	
Diodes, Transistors and Photocells	
Electronic Microcircuits	
Optical Lenses	
Parts of Metalworking Machine Tools	Automotive Electrical Equipment
Circuit Breakers and Panels	
Non-Electrical Counting Devices	
Control Instruments of Gas or Liquid	
Miscellaneous Engines	Vehicles Parts and Accessories
Parts of Metalworking Machine Tools	
Miscellaneous Metalworking Machinery	
Circuit Breakers and Panels	

## Appendix A. “Core Products” and Their Neighbors

**Glycosides and Vaccines:**

Amine-Function Compounds, Inorganic Esters, Hormones, Non-Medicinal Pharmaceutical Products, Chemical Products, Printing Machine Parts, Furnaces, Analog Instruments for Physical Analysis, Orthopedic Devices.

**Motor Vehicles Piston Engines:**

Pig Meat, Miscellaneous Rubber, Miscellaneous Refractory Goods, Worked Nickel, Metal Springs, Piston Engine Parts, Rotary Pumps, Miscellaneous Liquid Pump Parts, Miscellaneous Centrifuge and Filtering Machinery, Pulley System Parts, Automotive Electrical Equipment, Vehicle Bodies, Vehicles Parts and Accessories, Non-Electrical Counting Devices, Control Instruments of Gas or Liquid.

**Piston Engine Parts:**

Miscellaneous Rubber, Abrasive Powder, Miscellaneous Refractory Goods, Locksmith Hardware, Metal Springs, Miscellaneous Parts of Steam Power Units, Motor Vehicles Piston Engines, Tractors, Rolling Mills, Industrial Furnaces and Ovens, Reciprocating Pumps, Air Pumps and Compressors, Miscellaneous Pump Parts, Filtering and Purifying Machinery, Miscellaneous Centrifuge and Filtering Machinery, Valves, Pulley System Parts, Electrical Insulators, Automotive Electrical Equipment, Vehicles Parts and Accessories, Control Instruments of Gas or Liquid.

**Machinery for Specialized Industries:**

Interchangeable Tool Parts, Tool Holders, Miscellaneous Metalworking Machinery, Furnaces, Mathematical Calculation Instruments, Analog Instruments for Physical Analysis.

**Miscellaneous Heating and Cooling Equipment:**

Abrasive Powder, Interchangeable Tool Parts, Miscellaneous Engines, Paper Making Machine Parts, Printing Machine Parts, Miscellaneous Metalworking Machinery, Furnaces, Industrial Furnaces and Ovens, Centrifugal Pumps, Rotary Pumps, Miscellaneous Liquid Pump Parts, Miscellaneous Centrifuge and Filtering Machinery, Factory Trucks, Lifting and Loading Machinery, Miscellaneous Non-Electrical Machines, Valves, Pulley System Parts, Miscellaneous Non-Electrical Machinery Parts.

**Pulley System Parts:**

Polyamides, Miscellaneous Rubber, Iron Sheets, Interchangeable Tool Parts, Metal Springs, Miscellaneous Parts of Steam Power Units, Motor Vehicles Piston Engines, Piston Engine Parts, Miscellaneous Engines, Harvesting Machines, Auxiliary Weaving Machinery, Paper Making Machine Parts, Tool Holders, Parts of Metalworking Machine Tools, Miscellaneous Metalworking Machinery, Industrial Furnaces and Ovens, Miscellaneous Heating and Cooling Equipment, Rotary Pumps, Miscellaneous Liquid Pump Parts, Air Pumps and Compressors, Miscellaneous Pump Parts, Miscellaneous Centrifuge and Filtering Machinery, Roller Bearings, Valves, Electrical Insulators, Automotive Electrical Equipment, Vehicle Bodies, Vehicles Parts and Accessories, Railway Track Fixtures and Fittings.

**Miscellaneous Office Equipment:**

Video and Sound Recorders.

**CPUs:**

Computer Peripherals, Miscellaneous Data Processing Equipment.

**Computer Peripherals:**

CPUs, Miscellaneous Data Processing Equipment, Computer Parts and Accessories, Vehicles Stereos, Video and Sound Recorders, Telecom Parts and Accessories, Printed Circuits, Diodes, Transistors and Photocells, Miscellaneous Electronic Circuit Parts, Optical Lense.

**Computer Parts and Accessories:**

Epoxide Resins, Computer Peripherals, Miscellaneous Data Processing Equipment, Vehicles Stereos, Printed Circuits, Diodes, Transistors and Photocells, Electronic Microcircuits, Miscellaneous Electronic Circuit Parts, Miscellaneous Electrical Machinery, Optical Lenses.

**Color TVs:**

Mirrors, Air Conditioners, Vehicles Stereos, Washing Machines.

**Telecom Parts and Accessories:**

Computer Peripherals, Vehicles Stereos, Telephone Line, TV and Radio Transmitters, Miscellaneous Power Machinery, Printed Circuits, Miscellaneous Electrical Machinery.

**Printed Circuits:**

Computer Peripherals, Computer Parts and Accessories, Telecom Parts and Accessories, Diodes, Transistors and Photocells, Electronic Microcircuits, Miscellaneous Electronic Circuit Parts, Optical Lenses, Clocks.

**Miscellaneous Electronic Circuit Parts:**

Computer Peripherals, Computer Parts and Accessories, Printed Circuits, Diodes, Transistors and Photocells, Electronic Microcircuits, Optical Lenses.

**Automotive Electrical Equipment:**

Aminoplasts, Miscellaneous Rubber, Locksmith Hardware, Metal Springs, Miscellaneous Parts of Steam Power Units, Motor Vehicles Piston Engines, Piston Engine Parts, Parts of Metalworking Machine Tools, Air Pumps and Compressors, Miscellaneous Pump Parts, Valves, Pulley System Parts, Circuit Breakers and Panels, Electrical Insulators, Vehicles Parts and Accessories, Non-Electrical Counting Devices, Control Instruments of Gas or Liquid.

**Vehicles Parts and Accessories:**

Aminoplasts, Car Tires, Transmission Belts, Miscellaneous Rubber, Miscellaneous Paper, Refractory Bricks, Abrasive Powder, Safety Glass, Iron Sheets, Seamless Iron Tubes, Iron and Steel Forging, Locksmith Hardware, Metal Springs, Miscellaneous Articles of Iron, Miscellaneous Metal Articles, Miscellaneous Parts of Steam Power Units, Motor Vehicles Piston Engines, Piston Engine Parts, Miscellaneous Rotating Electric Plant Parts, Miscellaneous Engines, Harvesting Machines, Miscellaneous Textile Machinery, Parts of Metalworking Machine Tools, Rolling Mills, Miscellaneous Metalworking Machinery, Industrial Furnaces and Ovens, Miscellaneous Refrigeration Equipment, Miscellaneous Liquid Pump Parts, Air Pumps and Compressors, Filtering and Purifying Machinery, Lifting and Loading Machinery, Miscellaneous Parts of Lifting Machinery, Valves, Pulley System Parts, Miscellaneous Non-Electrical Machinery Parts, Circuit Breakers and Panels, Electrical Insulators, Automotive Electrical Equipment, Trucks and Vans, Railway Track Fixtures and Fittings, Central Heating Equipment.

**Lighting Fixtures:**

Home Electrical Appliances.

**Optical Lenses:**

Computer Peripherals, Computer Parts and Accessories, Printed Circuits, Diodes, Transistors and Photocells, Electronic Microcircuits, Miscellaneous Electronic Circuit Parts.
